# *catena*-Poly[[aqua­(dipyrido[3,2-*a*:2′,3′-*c*]phenazine-κ^2^ *N* ^4^,*N* ^5^)iron(II)]-μ-pyrazine-2,3-dicarboxyl­ato-κ^3^ *N* ^1^,*O* ^2^:*O* ^3^]

**DOI:** 10.1107/S1600536808027153

**Published:** 2008-09-06

**Authors:** Zhan-Lin Xu, Xiu-Ying Li, Guang-Bo Che, Lu Lu, Chun-Hui Xu

**Affiliations:** a Department of Chemistry, Jilin Normal University, Siping 136000, People’s Republic of China

## Abstract

In the title compound, [Fe(C_6_H_2_N_2_O_4_)(C_18_H_10_N_4_)(H_2_O)]_*n*_, the Fe^II^ ion adopts a slightly distorted octahedral *mer*-FeN_3_O_3_ geometry, arising from one *N*,*N*′-bidentate dipyrido[3,2-*a*:2′,3′-*c*]phenazine ligand, one *N*,*O*-chelating pyrazine-2,3-dicarboxyl­ate dianion and one water mol­ecule. An O-bonded symmetry-related dianion completes the coordination of the metal. The bridging dianion results in a one-dimensional polymeric chain. Aromatic π–π stacking inter­actions between ligands [centroid–centroid separations = 3.528 (2) and 3.741 (2) Å] and O—H⋯O and O—H⋯N hydrogen bonds link the chains together, leading to a three-dimensional supra­molecular network.

## Related literature

For related literature, see: Che *et al.* (2006 ▶); Stephenson & Hardie (2006 ▶); Wang *et al.* (2008 ▶); Xu *et al.* (2008 ▶).
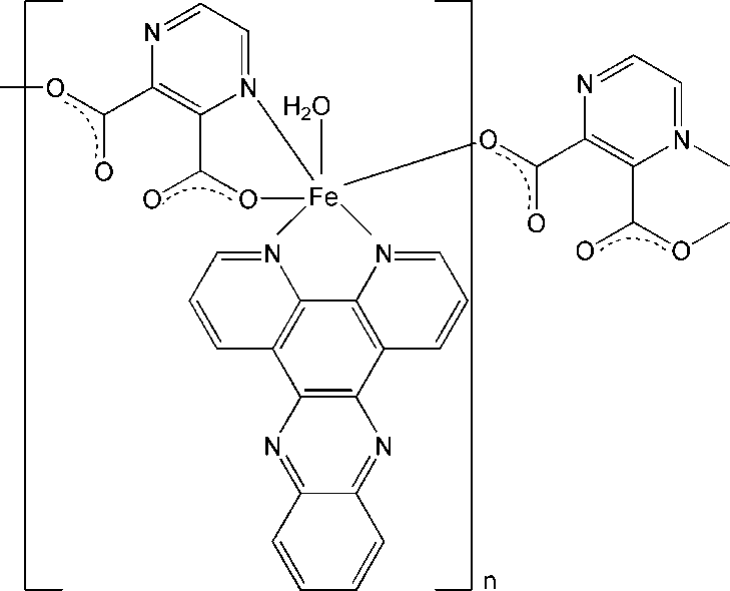


## Experimental

### 

#### Crystal data

[Fe(C_6_H_2_N_2_O_4_)(C_18_H_10_N_4_)(H_2_O)]
*M* *_r_* = 522.26
Triclinic, 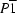

*a* = 6.7868 (14) Å
*b* = 7.4586 (15) Å
*c* = 20.548 (4) Å
α = 90.75 (3)°
β = 95.89 (3)°
γ = 98.54 (3)°
*V* = 1022.8 (4) Å^3^
*Z* = 2
Mo *K*α radiation
μ = 0.79 mm^−1^
*T* = 292 (2) K
0.35 × 0.30 × 0.25 mm

#### Data collection

Rigaku R-AXIS RAPID diffractometer
Absorption correction: multi-scan (*ABSCOR*; Higashi, 1995 ▶) *T*_min_ = 0.756, *T*_max_ = 0.821
10101 measured reflections
4633 independent reflections
2914 reflections with *I* > 2σ(*I*)
*R*_int_ = 0.056

#### Refinement

*R*[*F* ^2^ > 2σ(*F* ^2^)] = 0.056
*wR*(*F* ^2^) = 0.129
*S* = 1.04
4633 reflections
333 parameters
H atoms treated by a mixture of independent and constrained refinement
Δρ_max_ = 0.47 e Å^−3^
Δρ_min_ = −0.45 e Å^−3^

### 

Data collection: *PROCESS-AUTO* (Rigaku, 1998 ▶); cell refinement: *PROCESS-AUTO*; data reduction: *PROCESS-AUTO*; program(s) used to solve structure: *SHELXS97* (Sheldrick, 2008 ▶); program(s) used to refine structure: *SHELXL97* (Sheldrick, 2008 ▶); molecular graphics: *SHELXTL-Plus* (Sheldrick, 2008 ▶); software used to prepare material for publication: *SHELXL97*.

## Supplementary Material

Crystal structure: contains datablocks global, I. DOI: 10.1107/S1600536808027153/hb2771sup1.cif

Structure factors: contains datablocks I. DOI: 10.1107/S1600536808027153/hb2771Isup2.hkl

Additional supplementary materials:  crystallographic information; 3D view; checkCIF report

## Figures and Tables

**Table 1:** Selected bond lengths (Å)

Fe—N5	2.160 (3)
Fe—N2	2.172 (3)
Fe—N1	2.193 (3)
Fe—O3^i^	2.042 (3)
Fe—O1	2.113 (3)
Fe—O1*W*	2.148 (3)

Symmetry code: (i) 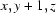.

**Table 2:** Hydrogen-bond geometry (Å, °)

*D*—H⋯*A*	*D*—H	H⋯*A*	*D*⋯*A*	*D*—H⋯*A*
O1*W*—H*W*1*B*⋯O4^ii^	0.89 (7)	1.79 (7)	2.649 (4)	161 (5)
O1*W*—H*W*1*A*⋯N6^iii^	0.81 (5)	2.05 (5)	2.861 (4)	176 (5)

Symmetry codes: (ii) 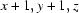; (iii) 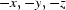.

**Fig. 1.**
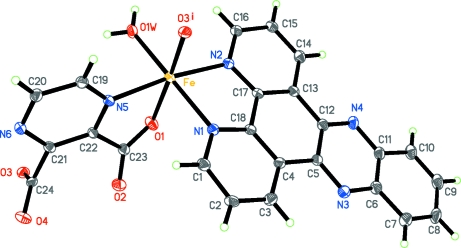
The asymmetric unit of (I), expanded to show the metal coordination sphere. Displacement ellipsoids are drawn at the 20% probability level (arbitrary spheres for the H atoms). [Symmetry code: (i) x, y + 1, z.]

**Fig. 2.**
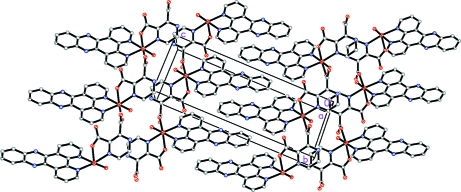
Packing diagram of the three-dimensional supramolecular structure of (I) formed viaπ-π interactions. H atoms have been omitted.

## supplementary crystallographic information

### Comment

Dipyrido[3,2-a:2',3'-c]phenazine (DPPZ) is an important derivative of 1,10-phenanthroline (phen), having often been used to build novel supramolecular architectures due to its excellent coordinating ability to metals and its rigid planar aromatic ring system (Stephenson & Hardie, 2006).  A few complexes containing DPPZ in combination with doubly-deprotonated pyrazine-2,3-dicarboxylic acid (H_2_PZDC) have been reported (Xu *et al.*, 2008; Wang *et al.*, 2008). As a continuation of this work, we selected H_2_PZDC as a linker ligand and DPPZ as a secondary chelating ligand, generating a new coordination polymer, [Fe(DPPZ)(PZDC)(H_2_O)]_n_, (I), which is reported here.

The Fe(II) atom is bonded to three nitrogen atoms (N1, N2, N5) from one DPPZ ligand and one PZDC ligand, and three oxygen atoms (O1, O3^i^, O1W) from two PZDC ligands and one water molecule in a slightly distorted octahedral coordination geometry (Table 1). The mean bond distances are Fe—N = 2.175 (3) Å and Fe—O = 2.101 (3) Å. The N1, N2, N5, O1W atoms comprise the basal plane, while the O1 and O3 atoms occupy the axial position (Fig. 1). The PZDC^2-^ dianion adopts chelating and bridging coordination modes, linking the adjacent Fe atoms into a distinctive one-dimensional chain propagating along the *b* axis, and the DPPZ ligands are attached to one side of the chain. The neighboring one-dimensional chains interact by π-π stacking between the dppz ligands [centroid separation = 3.741 (2) Å], at the same time, the π-π type interactions between two PZDC^2-^ ligands occur [centroid separation = 3.528 (2) Å]. In this way, these neighboring one-dimensional chains are linked into an intriguing three-dimensional supramolecular motif (Fig. 2). Furthermore, O—H···O and O—H···N hydrogen bonds between the O1W atom and the O4, N6 atoms of the PZDC^2-^ dianion further consolidate the three-dimensional architecture (Table 2).

### Experimental

The DPPZ ligand was synthesized according to the literature method (Che, Li *et al.*, 2006). A mixture of DPPZ, H_2_PZDC, FeSO_4_ and water in a molar ratio of 1:1:1:5000 was sealed in a Teflon-lined autoclave and heated to 433 K for 3 d. Upon cooling and opening the bomb, brown blocks of (I) were obtained (75% yield based on Fe).

### Refinement

All H atoms on C atoms were positioned geometrically (C—H = 0.93 Å) and refined as riding, with *U*_iso_(H)= 1.2*U*_eq_(C). The hydrogen atoms of water molecules were located from difference Fourier maps and their positions and *U*_iso_ values were refined freely.

### Crystal data

**Table:**

[Fe(C_6_H_2_N_2_O_4_)(C_18_H_10_N_4_)(H_2_O)]	*Z* = 2
*M**_r_* = 522.26	*F*(000) = 532
Triclinic, *P*1	*D*_x_ = 1.696 Mg m^−^^3^
Hall symbol: -P 1	Mo *K*α radiation, λ = 0.71073 Å
*a* = 6.7868 (14) Å	Cell parameters from 2687 reflections
*b* = 7.4586 (15) Å	θ = 3.0–27.5°
*c* = 20.548 (4) Å	µ = 0.79 mm^−^^1^
α = 90.75 (3)°	*T* = 292 K
β = 95.89 (3)°	Block, brown
γ = 98.54 (3)°	0.35 × 0.30 × 0.25 mm
*V* = 1022.8 (4) Å^3^	

### Data collection

**Table:**

Rigaku R-AXIS RAPID diffractometer	4633 independent reflections
Radiation source: fine-focus sealed tube	2914 reflections with *I* > 2σ(*I*)
graphite	*R*_int_ = 0.056
Detector resolution: 10.0 pixels mm^-1^	θ_max_ = 27.5°, θ_min_ = 3.0°
ω scans	*h* = −8→8
Absorption correction: multi-scan (ABSCOR; Higashi, 1995)	*k* = −9→8
*T*_min_ = 0.756, *T*_max_ = 0.821	*l* = −25→26
10101 measured reflections	

### Refinement

**Table:**

Refinement on *F*^2^	Primary atom site location: structure-invariant direct methods
Least-squares matrix: full	Secondary atom site location: difference Fourier map
*R*[*F*^2^ > 2σ(*F*^2^)] = 0.056	Hydrogen site location: difmap and geom
*wR*(*F*^2^) = 0.129	H atoms treated by a mixture of independent and constrained refinement
*S* = 1.04	*w* = 1/[σ^2^(*F*_o_^2^) + (0.0428*P*)^2^ + 1.0171*P*] where *P* = (*F*_o_^2^ + 2*F*_c_^2^)/3
4633 reflections	(Δ/σ)_max_ = 0.004
333 parameters	Δρ_max_ = 0.47 e Å^−^^3^
0 restraints	Δρ_min_ = −0.45 e Å^−^^3^

### Special details

**Table:**

Geometry. All e.s.d.'s (except the e.s.d. in the dihedral angle between two l.s. planes) are estimated using the full covariance matrix. The cell e.s.d.'s are taken into account individually in the estimation of e.s.d.'s in distances, angles and torsion angles; correlations between e.s.d.'s in cell parameters are only used when they are defined by crystal symmetry. An approximate (isotropic) treatment of cell e.s.d.'s is used for estimating e.s.d.'s involving l.s. planes.
Refinement. Refinement of *F*^2^ against ALL reflections. The weighted *R*-factor *wR* and goodness of fit *S* are based on *F*^2^, conventional *R*-factors *R* are based on *F*, with *F* set to zero for negative *F*^2^. The threshold expression of *F*^2^ > σ(*F*^2^) is used only for calculating *R*-factors(gt) *etc*. and is not relevant to the choice of reflections for refinement. *R*-factors based on *F*^2^ are statistically about twice as large as those based on *F*, and *R*- factors based on ALL data will be even larger.

### Fractional atomic coordinates and isotropic or equivalent isotropic displacement parameters (Å^2^)

**Table:**

	*x*	*y*	*z*	*U*_iso_*/*U*_eq_	
C1	−0.3579 (6)	0.3353 (5)	0.21577 (19)	0.0391 (10)	
H1	−0.4134	0.2742	0.1769	0.047*	
C2	−0.4714 (6)	0.3250 (5)	0.26882 (19)	0.0399 (10)	
H2	−0.5990	0.2575	0.2657	0.048*	
C3	−0.3893 (6)	0.4177 (5)	0.32583 (19)	0.0391 (9)	
H3	−0.4625	0.4147	0.3618	0.047*	
C4	−0.1974 (5)	0.5158 (5)	0.32993 (17)	0.0309 (8)	
C5	−0.1015 (6)	0.6154 (5)	0.38923 (17)	0.0324 (8)	
C6	−0.1076 (7)	0.7026 (5)	0.49600 (18)	0.0410 (10)	
C7	−0.2116 (8)	0.7065 (6)	0.5526 (2)	0.0536 (12)	
H7	−0.3433	0.6493	0.5517	0.064*	
C8	−0.1156 (8)	0.7952 (6)	0.6082 (2)	0.0563 (13)	
H8	−0.1829	0.7977	0.6454	0.068*	
C9	0.0830 (8)	0.8825 (6)	0.6102 (2)	0.0545 (13)	
H9	0.1454	0.9415	0.6487	0.065*	
C10	0.1856 (7)	0.8821 (6)	0.55679 (19)	0.0478 (11)	
H10	0.3172	0.9404	0.5588	0.057*	
C11	0.0912 (7)	0.7924 (5)	0.49774 (18)	0.0391 (10)	
C12	0.0969 (6)	0.7099 (5)	0.39062 (18)	0.0350 (9)	
C13	0.2008 (6)	0.7143 (5)	0.33132 (17)	0.0324 (8)	
C14	0.3877 (6)	0.8170 (5)	0.3279 (2)	0.0431 (10)	
H14	0.4520	0.8862	0.3640	0.052*	
C15	0.4771 (6)	0.8157 (5)	0.2708 (2)	0.0422 (10)	
H15	0.5998	0.8873	0.2673	0.051*	
C16	0.3807 (6)	0.7056 (5)	0.21845 (19)	0.0370 (9)	
H16	0.4438	0.7016	0.1805	0.044*	
C17	0.1100 (6)	0.6141 (5)	0.27547 (17)	0.0305 (8)	
C18	−0.0933 (5)	0.5160 (5)	0.27466 (17)	0.0293 (8)	
C19	−0.2089 (6)	0.2259 (5)	0.01794 (18)	0.0336 (9)	
H19	−0.2207	0.3400	0.0016	0.040*	
C20	−0.2698 (6)	0.0736 (5)	−0.02246 (18)	0.0365 (9)	
H20	−0.3164	0.0881	−0.0660	0.044*	
C21	−0.1979 (5)	−0.1099 (5)	0.06260 (18)	0.0303 (8)	
C22	−0.1288 (5)	0.0440 (5)	0.10284 (17)	0.0285 (8)	
C23	−0.0384 (6)	0.0397 (5)	0.17379 (18)	0.0346 (9)	
C24	−0.2043 (5)	−0.2964 (5)	0.08552 (16)	0.0297 (8)	
N1	−0.1746 (5)	0.4280 (4)	0.21800 (14)	0.0331 (7)	
N2	0.2019 (5)	0.6055 (4)	0.22036 (14)	0.0314 (7)	
N3	−0.2043 (5)	0.6123 (4)	0.44118 (15)	0.0411 (8)	
N4	0.1938 (5)	0.7950 (4)	0.44469 (15)	0.0392 (8)	
N5	−0.1343 (5)	0.2118 (4)	0.07921 (14)	0.0305 (7)	
N6	−0.2634 (5)	−0.0942 (4)	−0.00061 (15)	0.0352 (8)	
O1	0.0834 (4)	0.1821 (3)	0.19258 (13)	0.0424 (7)	
O2	−0.0907 (5)	−0.0942 (4)	0.20571 (14)	0.0510 (8)	
O1W	0.2777 (5)	0.4031 (4)	0.08593 (15)	0.0452 (8)	
O3	−0.0399 (4)	−0.3617 (3)	0.09209 (12)	0.0375 (6)	
O4	−0.3708 (5)	−0.3852 (4)	0.09181 (15)	0.0550 (9)	
Fe	0.03575 (9)	0.42245 (7)	0.14403 (3)	0.03090 (17)	
HW1B	0.402 (10)	0.456 (8)	0.081 (3)	0.10 (2)*	
HW1A	0.279 (7)	0.315 (7)	0.063 (2)	0.056 (15)*	

### Atomic displacement parameters (Å^2^)

**Table:**

	*U*^11^	*U*^22^	*U*^33^	*U*^12^	*U*^13^	*U*^23^
C1	0.039 (2)	0.038 (2)	0.036 (2)	−0.0039 (18)	−0.0006 (17)	−0.0008 (18)
C2	0.030 (2)	0.041 (2)	0.044 (2)	−0.0067 (18)	0.0026 (17)	0.0041 (19)
C3	0.041 (2)	0.044 (2)	0.033 (2)	0.0053 (19)	0.0061 (17)	0.0035 (18)
C4	0.029 (2)	0.0298 (19)	0.0337 (19)	0.0028 (16)	0.0041 (16)	0.0019 (16)
C5	0.040 (2)	0.0282 (19)	0.0289 (18)	0.0044 (16)	0.0051 (16)	0.0016 (16)
C6	0.056 (3)	0.037 (2)	0.031 (2)	0.011 (2)	0.0028 (18)	0.0013 (17)
C7	0.070 (3)	0.051 (3)	0.041 (2)	0.007 (2)	0.015 (2)	0.000 (2)
C8	0.090 (4)	0.051 (3)	0.032 (2)	0.021 (3)	0.015 (2)	−0.005 (2)
C9	0.085 (4)	0.045 (3)	0.033 (2)	0.015 (3)	−0.002 (2)	−0.006 (2)
C10	0.059 (3)	0.046 (3)	0.036 (2)	0.010 (2)	−0.005 (2)	−0.008 (2)
C11	0.053 (3)	0.033 (2)	0.031 (2)	0.0098 (19)	0.0004 (18)	−0.0010 (17)
C12	0.041 (2)	0.0308 (19)	0.0328 (19)	0.0059 (17)	0.0011 (17)	0.0008 (16)
C13	0.035 (2)	0.0278 (18)	0.0330 (19)	−0.0015 (16)	0.0030 (16)	−0.0010 (16)
C14	0.043 (3)	0.040 (2)	0.042 (2)	−0.0042 (19)	0.0025 (19)	−0.0093 (19)
C15	0.038 (2)	0.036 (2)	0.049 (2)	−0.0087 (18)	0.0076 (19)	−0.0047 (19)
C16	0.035 (2)	0.034 (2)	0.042 (2)	−0.0001 (17)	0.0087 (17)	0.0003 (18)
C17	0.030 (2)	0.0255 (18)	0.0344 (19)	−0.0007 (15)	0.0022 (15)	0.0026 (15)
C18	0.029 (2)	0.0276 (18)	0.0294 (18)	0.0002 (15)	0.0014 (15)	0.0032 (15)
C19	0.034 (2)	0.0312 (19)	0.036 (2)	0.0045 (16)	0.0067 (17)	0.0019 (16)
C20	0.036 (2)	0.042 (2)	0.0302 (19)	0.0026 (18)	0.0027 (16)	−0.0011 (17)
C21	0.0236 (19)	0.0274 (18)	0.040 (2)	0.0028 (15)	0.0058 (16)	−0.0001 (16)
C22	0.028 (2)	0.0237 (17)	0.0336 (19)	0.0007 (15)	0.0061 (15)	−0.0034 (15)
C23	0.039 (2)	0.030 (2)	0.0343 (19)	0.0043 (17)	0.0038 (17)	0.0022 (17)
C24	0.025 (2)	0.038 (2)	0.0240 (17)	−0.0043 (16)	0.0053 (15)	−0.0002 (15)
N1	0.0338 (18)	0.0331 (16)	0.0300 (15)	−0.0017 (14)	0.0028 (13)	−0.0009 (14)
N2	0.0305 (18)	0.0305 (16)	0.0324 (16)	0.0011 (13)	0.0048 (13)	0.0000 (13)
N3	0.050 (2)	0.0378 (19)	0.0347 (17)	0.0026 (16)	0.0059 (16)	0.0009 (15)
N4	0.046 (2)	0.0376 (18)	0.0330 (17)	0.0071 (16)	0.0007 (15)	−0.0060 (15)
N5	0.0311 (18)	0.0252 (15)	0.0344 (16)	0.0002 (13)	0.0062 (13)	−0.0005 (13)
N6	0.0367 (19)	0.0315 (17)	0.0354 (17)	−0.0023 (14)	0.0055 (14)	−0.0050 (14)
O1	0.0450 (17)	0.0328 (15)	0.0445 (16)	0.0000 (13)	−0.0099 (13)	0.0003 (13)
O2	0.068 (2)	0.0383 (16)	0.0451 (16)	0.0004 (15)	0.0098 (15)	0.0096 (14)
O1W	0.0364 (18)	0.0424 (17)	0.0549 (18)	−0.0070 (14)	0.0172 (14)	−0.0195 (15)
O3	0.0453 (18)	0.0302 (14)	0.0388 (14)	0.0069 (12)	0.0114 (13)	0.0064 (12)
O4	0.047 (2)	0.0442 (17)	0.068 (2)	−0.0158 (15)	0.0120 (16)	0.0024 (15)
Fe	0.0342 (3)	0.0253 (3)	0.0314 (3)	−0.0014 (2)	0.0042 (2)	−0.0020 (2)

### Geometric parameters (Å, °)

**Table:**

C1—N1	1.326 (5)	C15—H15	0.9300
C1—C2	1.395 (5)	C16—N2	1.331 (5)
C1—H1	0.9300	C16—H16	0.9300
C2—C3	1.375 (5)	C17—N2	1.353 (4)
C2—H2	0.9300	C17—C18	1.461 (5)
C3—C4	1.390 (5)	C18—N1	1.353 (4)
C3—H3	0.9300	C19—N5	1.319 (5)
C4—C18	1.398 (5)	C19—C20	1.384 (5)
C4—C5	1.460 (5)	C19—H19	0.9300
C5—N3	1.332 (5)	C20—N6	1.340 (5)
C5—C12	1.421 (5)	C20—H20	0.9300
C6—N3	1.360 (5)	C21—N6	1.341 (5)
C6—C11	1.412 (6)	C21—C22	1.399 (5)
C6—C7	1.423 (6)	C21—C24	1.471 (5)
C7—C8	1.368 (6)	C22—N5	1.353 (4)
C7—H7	0.9300	C22—C23	1.525 (5)
C8—C9	1.403 (7)	C23—O2	1.230 (4)
C8—H8	0.9300	C23—O1	1.274 (4)
C9—C10	1.360 (6)	C24—O4	1.242 (4)
C9—H9	0.9300	C24—O3	1.278 (5)
C10—C11	1.422 (5)	Fe—N5	2.160 (3)
C10—H10	0.9300	Fe—N2	2.172 (3)
C11—N4	1.351 (5)	Fe—N1	2.193 (3)
C12—N4	1.333 (5)	Fe—O3^i^	2.042 (3)
C12—C13	1.468 (5)	Fe—O1	2.113 (3)
C13—C14	1.390 (5)	Fe—O1W	2.148 (3)
C13—C17	1.397 (5)	O1W—HW1B	0.89 (7)
C14—C15	1.375 (6)	O1W—HW1A	0.81 (5)
C14—H14	0.9300	O3—Fe^ii^	2.042 (3)
C15—C16	1.391 (5)		
			
N1—C1—C2	123.2 (3)	N1—C18—C17	116.7 (3)
N1—C1—H1	118.4	C4—C18—C17	121.0 (3)
C2—C1—H1	118.4	N5—C19—C20	121.1 (4)
C3—C2—C1	118.0 (4)	N5—C19—H19	119.5
C3—C2—H2	121.0	C20—C19—H19	119.5
C1—C2—H2	121.0	N6—C20—C19	121.8 (4)
C2—C3—C4	120.2 (4)	N6—C20—H20	119.1
C2—C3—H3	119.9	C19—C20—H20	119.1
C4—C3—H3	119.9	N6—C21—C22	120.8 (3)
C3—C4—C18	117.8 (3)	N6—C21—C24	115.7 (3)
C3—C4—C5	122.8 (3)	C22—C21—C24	123.5 (3)
C18—C4—C5	119.4 (3)	N5—C22—C21	120.4 (3)
N3—C5—C12	122.0 (3)	N5—C22—C23	115.0 (3)
N3—C5—C4	118.1 (3)	C21—C22—C23	124.6 (3)
C12—C5—C4	120.0 (3)	O2—C23—O1	128.1 (4)
N3—C6—C11	121.7 (4)	O2—C23—C22	118.3 (3)
N3—C6—C7	118.6 (4)	O1—C23—C22	113.5 (3)
C11—C6—C7	119.7 (4)	O4—C24—O3	124.1 (4)
C8—C7—C6	119.2 (5)	O4—C24—C21	117.9 (4)
C8—C7—H7	120.4	O3—C24—C21	117.6 (3)
C6—C7—H7	120.4	C1—N1—C18	118.4 (3)
C7—C8—C9	121.0 (4)	C1—N1—Fe	126.7 (2)
C7—C8—H8	119.5	C18—N1—Fe	114.2 (2)
C9—C8—H8	119.5	C16—N2—C17	118.2 (3)
C10—C9—C8	121.0 (4)	C16—N2—Fe	127.2 (2)
C10—C9—H9	119.5	C17—N2—Fe	114.6 (2)
C8—C9—H9	119.5	C5—N3—C6	116.2 (4)
C9—C10—C11	119.8 (4)	C12—N4—C11	116.6 (4)
C9—C10—H10	120.1	C19—N5—C22	118.3 (3)
C11—C10—H10	120.1	C19—N5—Fe	128.0 (3)
N4—C11—C6	121.6 (3)	C22—N5—Fe	112.6 (2)
N4—C11—C10	119.3 (4)	C20—N6—C21	117.4 (3)
C6—C11—C10	119.1 (4)	C23—O1—Fe	116.1 (2)
N4—C12—C5	121.9 (3)	Fe—O1W—HW1B	141 (4)
N4—C12—C13	118.3 (3)	Fe—O1W—HW1A	122 (3)
C5—C12—C13	119.8 (3)	HW1B—O1W—HW1A	97 (5)
C14—C13—C17	117.8 (3)	C24—O3—Fe^ii^	130.2 (2)
C14—C13—C12	122.5 (3)	O3^i^—Fe—O1	173.46 (11)
C17—C13—C12	119.7 (3)	O3^i^—Fe—O1W	90.86 (12)
C15—C14—C13	119.7 (4)	O1—Fe—O1W	91.40 (13)
C15—C14—H14	120.1	O3^i^—Fe—N5	97.15 (12)
C13—C14—H14	120.1	O1—Fe—N5	76.90 (11)
C14—C15—C16	118.8 (4)	O1W—Fe—N5	85.84 (12)
C14—C15—H15	120.6	O3^i^—Fe—N2	90.36 (11)
C16—C15—H15	120.6	O1—Fe—N2	95.42 (11)
N2—C16—C15	122.8 (4)	O1W—Fe—N2	97.75 (12)
N2—C16—H16	118.6	N5—Fe—N2	171.65 (12)
C15—C16—H16	118.6	O3^i^—Fe—N1	97.51 (12)
N2—C17—C13	122.5 (3)	O1—Fe—N1	80.95 (12)
N2—C17—C18	117.4 (3)	O1W—Fe—N1	169.62 (13)
C13—C17—C18	120.0 (3)	N5—Fe—N1	99.12 (11)
N1—C18—C4	122.3 (3)	N2—Fe—N1	76.17 (11)

Symmetry codes: (i) *x*, *y*+1, *z*; (ii) *x*, *y*−1, *z*.

### Hydrogen-bond geometry (Å, °)

**Table:**

*D*—H···*A*	*D*—H	H···*A*	*D*···*A*	*D*—H···*A*
O1W—HW1B···O4^iii^	0.89 (7)	1.79 (7)	2.649 (4)	161 (5)
O1W—HW1A···N6^iv^	0.81 (5)	2.05 (5)	2.861 (4)	176 (5)

Symmetry codes: (iii) *x*+1, *y*+1, *z*; (iv) −*x*, −*y*, −*z*.
